# Anti-apoptotic and pro-survival effect of exercise training on early aged hypertensive rat cerebral cortex

**DOI:** 10.18632/aging.203431

**Published:** 2021-08-25

**Authors:** Yi-Jie Liu, Zhen-Yang Cui, Ai-Lun Yang, Amadou W. Jallow, Hai-Liang Huang, Chun-Lei Shan, Shin-Da Lee

**Affiliations:** 1 School of Rehabilitation Medicine, Shanghai University of Traditional Chinese Medicine, Shanghai, China; 2 Institute of Rehabilitation Medicine, Shanghai University of Traditional Chinese Medicine, Shanghai, China; 3 School of Rehabilitation Medicine, Weifang Medical University, Shandong, China; 4 Institute of Sports Sciences, University of Taipei, Taipei, Taiwan; 5 Department of Medical Laboratory and Biotechnology, Asia University, Taichung, Taiwan; 6 College of Rehabilitation, Shandong University of Traditional Chinese Medicine, Shandong, China; 7 Department of Physical Therapy, Graduate Institute of Rehabilitation Science, China Medical University, Taichung, Taiwan; 8 Department of Physical Therapy, Asia University, Taichung, Taiwan

**Keywords:** brain, early aged hypertension, neuroprotection, neuronal apoptosis

## Abstract

The anti-apoptotic and pro-survival effects of exercise training were evaluated on the early aged hypertensive rat cerebral cortex. The brain tissues were analysed from ten sedentary male Wistar Kyoto normotensive rats (WKY), ten sedentary spontaneously 12 month early aged hypertensive rats (SHR), and ten hypertensive rats undergoing treadmill exercise training (60 min/day, 5 days/week) for 12 weeks (SHR-EX). TUNEL-positive apoptotic cells, the expression levels of endonuclease G (EndoG) and apoptosis-inducing factor (AIF) (caspase-independent apoptotic pathway), Fas ligand, Fas death receptor, tumor necrosis factor (TNF)-α, TNF receptor 1, Fas-associated death domain, active caspase-8 and active caspase-3 (Fas-mediated apoptotic pathways) as well as t-Bid, Bax, Bak, Bad, cytochrome c, active caspase 9 and active caspase-3 (mitochondria-mediated apoptotic pathways) were reduced in SHR-EX compared with SHR. Pro-survival Bcl2, Bcl-xL, p-Bad, 14-3-3, insulin-like growth factor (IGF)-1, pPI3K/PI3K, and pAKT/AKT were significantly increased in SHR-EX compared to those in SHR. Exercise training suppressed neural EndoG/AIF-related caspase-independent, Fas/FasL-mediated caspase-dependent, mitochondria-mediated caspase-dependent apoptotic pathways as well as enhanced Bcl-2 family-related and IGF-1-related pro-survival pathways in the early aged hypertensive cerebral cortex. These findings indicated new therapeutic effects of exercise training on preventing early aged hypertension-induced neural apoptosis in cerebral cortex.

## INTRODUCTION

Hypertension (HTN) is considered as the most prevalent and leading cause of most morbidities and mortalities in worldwide health [1]. Progressive brain damage is one of the adverse complications of hypertension [2]. A previous study revealed that hypertension-induced structural changes in the brain such as vascular remodeling, cerebral microbleeds, cerebral atrophy, and impaired cerebral autoregulation [3]. Some studies reported that chronic hypertension appeared to aggravate Parkinson’s diseases, Alzheimer’s diseases, dementia, and cognitive impairment [4, 5]. Cerebral cortex apoptosis was found in a transgenic mouse model of Alzheimer's disease with cognitive dysfunction [6]. Neuroinflammation which was augmented in hypertension was also identified as a key determinant resulting in brain dysfunction [7]. Pro-inflammatory tumor necrosis factor (TNF) alpha also enhances neural cellular apoptotic pathway through binding tumor necrosis factor receptor 1 (TNFR1), a death receptor and harboring a death domain [8]. Moreover, high blood pressure deteriorates neuronal functions [9], and causes destruction on both small and large cerebral vessels leading to dementia and brain damage [10]. Therefore, strategies to control blood pressure would provide neurological benefits.

Exercise training had been known to lower the sympathetic activity and was beneficial for the prevention and treatment of hypertension and its cognitive impairments [11]. A study showed that aerobic exercise manifested both short-term and long-term effects on blood pressure control [12]. Exercise had demonstrated a neuroprotective effect in extensive studies and was recommended to counteract brain dysfunction in neurodegenerative disorders [13, 14]. Moreover, some studies revealed that chronic exercise training improved cognitive impairment as well as delayed the onset of neurodegenerative diseases including Alzheimer's disease and dementia [15, 16]. There was various overwhelming literature confirming that exercise training improved the brain cortex and cerebellum AIF function as well as reduced oxidative stress and apoptotic related markers [16, 17]. Decreased apoptotic neuronal cell death in the motor cortex was observed after exercise training which inhibited brain inflammation-induced motor impairment [18]. Pre-exercise training in traumatic brain areas significantly diminished lesion area, prevented neuronal loss, and reduced microglial activation in the cortex [19]. Numerous studies showed that voluntary running alone was adequate to enhance cell proliferation and survival, upgrade synaptic versatility and improve cognitive performance [20, 21]. However, the protective mechanism of exercise training on the cerebral cortex remains elusive.

Apoptosis is a form of programmed cell death and is regulated by diverse signalling pathways. However, the mechanism of neural apoptosis in cerebral cortex is complicated. Endonuclease G (EndoG) and apoptosis-inducing factor (AIF) caspase-independence apoptotic pathway is one of the mechanisms responsible for initiating neural apoptosis [22]. The initiation of this pathway involves the release of Endo G and AIF from the mitochondria to translocate into the nucleus. The Endo G is responsible to trigger DNA fragmentation [23] whereas AIF causes nuclear changes and chromatin condensation [24]. Previous studies had shown that exercise training significantly reduced AIF expression and interrupted AIF migration to the nucleus in brain injury [19, 25]. Currently, the effect of exercise training on Endo G/AIF neural cell death pathway remains unknown in the cerebral cortex of hypertension.

Extrinsic or Fas/FasL-initiated apoptotic pathway and intrinsic mitochondrial initiated apoptotic pathway were considered as the main mechanism in neural apoptosis [26]. Research has shown that Fas ligand binding to Fas receptor or TNF-α binding with TNFR1 receptors, death receptor Fas or tumor necrosis factor (TNF) receptors is directly involved in initiating Fas/FasL-mediated apoptotic pathway in neural apoptosis [27]. Initiation of the pathway involves activation of the death receptor Fas binding with Fas ligand and leads to the formation of death-inducing signalling complex (DISC). During the binding process of Fas receptor and Fas ligand, the adaptor protein Fas-associated death domain (FADD) and pro-caspase-8 are recruited and induce Caspase-8 activation. When caspase-8 is activated, it will subsequently activate Caspase-3 cleavage then induce apoptosis [26]. While the effect of exercise training on neural Fas-initiated apoptotic pathway in hypertensive cerebral cortex remains still unclear.

The mitochondria-mediated apoptotic pathway or intrinsic pathway in neural apoptosis involves the release of Cytochrome *c*. Interaction of either homodimers or heterodimers of Bcl-2 family proteins composing of both pro- and anti-apoptotic proteins leads to the release of Cytochrome *c* into the cytosol. Released Cytochrome c will interact with apoptosis protease activating factor-1 (Apaf-1) and pro-caspase-9 in the cytosol to form an apoptosome complex. By closed clustering and interacting with apoptosome, Cytochrome c leads to the activation of caspase-9 from pro-caspase-9. Then, activated caspase-9 will eventually cleave pro-caspase-3 to produce the effector protease caspase-3 and initiate apoptosis [28]. The action of pro-apoptotic proteins is strongly controlled by members of the anti-apoptotic Bcl-2 family proteins such as Bcl-2 and Bcl-xL and anti-apoptotic Bcl-2 family proteins such as Bcl-2, Bcl-xL, p-Bad, and 14-3-3 [29]. However, the role of exercise training on neural mitochondria-mediated apoptotic pathway in the hypertensive cerebral cortex is still unknown.

Insulin-like growth factor (IGF-1) plays an essential role in promoting cell proliferation, differentiation and inhibiting neural apoptosis through the activation of PI3K/AKT signaling pathway [30]. When IGF-1 activates phosphatidylinositol 3 kinase (PI3K), it will consequently activate the downstream of protein kinase B (AKT). The activation AKT promotes neural proliferation and regeneration [31, 32]. Phosphorylated AKT also interacts with Bcl-2 related pro-survival proteins to inhibit apoptosis [33].

The current study investigated whether exercise training could prevent neural apoptosis on early aged hypertensive cerebral cortex. Therefore, we hypothesized that exercise training might prevent hypertension-induced EndoG/AIF caspase-independent, Fas/FasL-mediated caspase-dependent and mitochondria-mediated caspase-dependent apoptotic pathways also aggrandized related Bcl-2 family and IGF-1 pro-survival pathways in the cerebral cortex.

## RESULTS

### Body weight and cortical characteristics

There were no significant differences in body weight and brain weight among the three groups (WKY, SHR, and SHR-EX). The heart rate, systolic, diastolic and mean arterial blood pressure were higher in SHR group than WKY group. The heart rate, systolic blood pressure, diastolic blood pressure and mean arterial blood pressure were significantly decreased in SHR-EX compared with SHR group. The recorded pulse pressure was higher in SHR group compared with WKY and was decreased in SHR-EX compared with SHR group. To determine the effectiveness of exercise training among three groups, we measured the Citrate Synthase Activity. The Citrate Synthase Activity in the SHE-EX group was higher compared with SHR or WKY (Table 1).

**Table 1:** Characteristics of WKY, SHR and SHR-EX groups.

	**WKY**	**SHR**	**SHR-EX**
Number of animals	10	10	10
Body weight, g	398 ± 9	402 ± 11	396±13
Brain weight, g	2.20±0.04	2.25±0.06	2.24±0.07
Heart rate, beats/min	279±11	390±17*	359±18*^#^
Systolic blood pressure, mmHg	114±4	193±5*	177±4*^#^
Diastolic blood pressure, mmHg	87±7	154±3*	136±4*^#^
Mean blood pressure, mmHg	95±6	167±3*	150±3*^#^
Pulse pressure, mmHg	27±3	39±2*	27±4*^#^
Citrate Synthase Activity (μmol min^-1^ g wet wt^-1^)	1.58±0.04	1.59±0.05	1.93±0.07*^#^

Values are means ± SEM amount a normotensive Wistar Kyoto (WKY) group, a spontaneously early aged hypertensive (SHR) group and a hypertension with 12 weeks Exercise training (SHR-EX) group. ^*^p<0.05 Significant differences between WKY and SHR or between WKY and SHR-EX group. ^#^*p*<0.05 significant differences between SHR group and SHR-EX group.

### TUNEL-positive apoptotic cells of cerebral cortex

**Figure 1:**
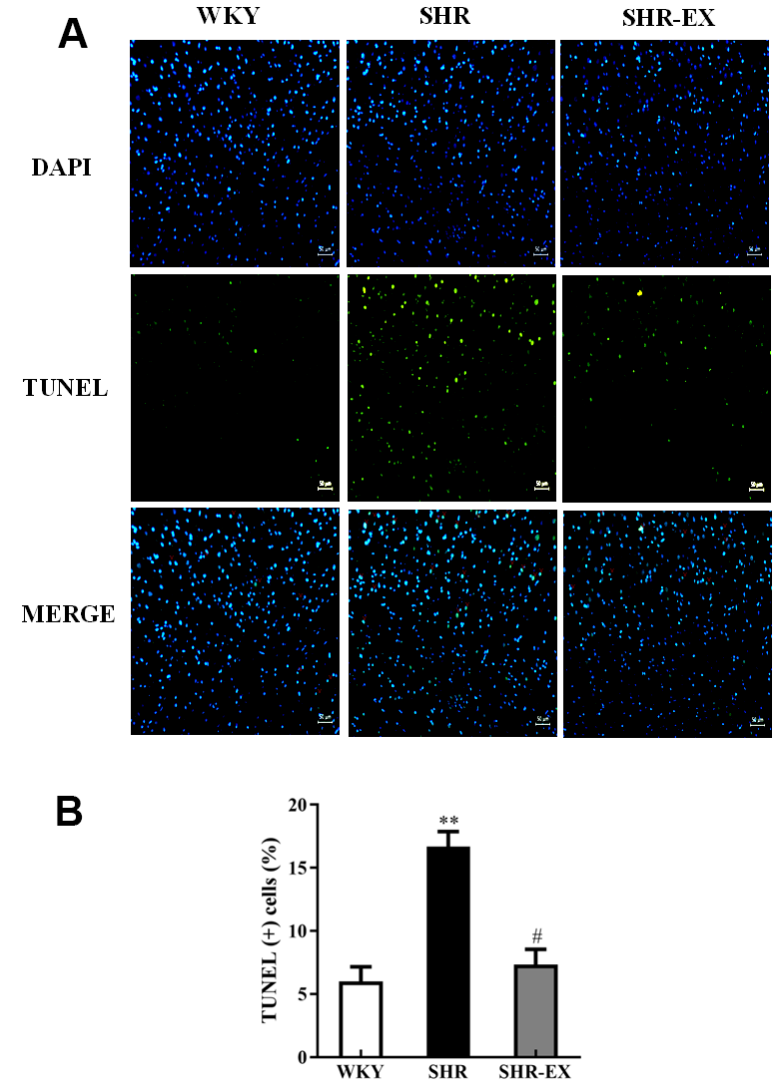
(**A**) Positive apoptotic neural cell was identified with 4′,6-diamidino-2-phenylindole (DAPI) staining and terminal deoxynucleotidyl transferase dUTP nick end labeling (TUNEL) assay. The upper image with blue spots indicates DAPI staining, middle image with green spots indicates TUNEL assay, and lower image with blue and green spots indicates Merge of DAPI staining and TUNEL assay. Scale bar, 50μm. (**B**) The bar shows the quantification of TUNEL apoptosis (%), determined as the proportion of TUNEL-positive cells relative to total DAPI stained nuclei. **: *p*<0.01 in comparison between SHR group and WKY rat group. #: *p*<0.05; in comparison between SHR-EX group and SHR group.

To determine the degree of apoptosis in the cerebral cortex of WKY, SHR, and SHR-EX after exercise training, TUNEL assay and DAPI staining were measured. We observed that the number of TUNEL-positive neural cells in the SHR group was greater than WKY, whereas, the number of TUNEL-positive neural cells in SHR-EX was lower than SHR group (Figure 1A, 1B).

### EndoG/AIF-related caspase-independent neural apoptotic pathway

**Figure 2:**
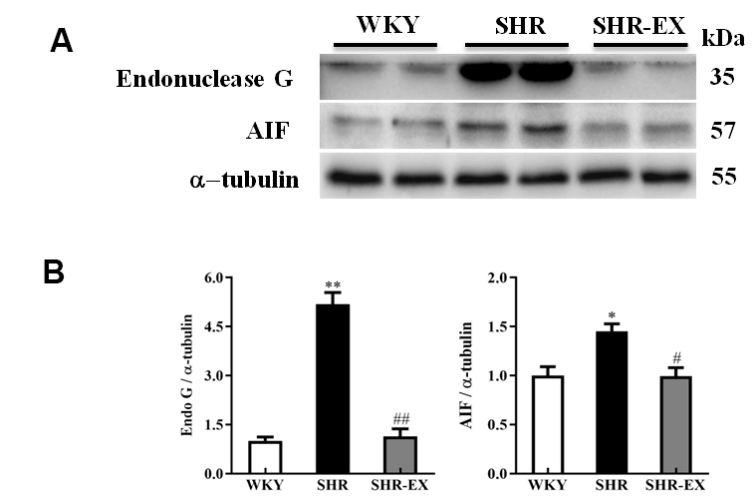
**The endonuclease G (EndoG) and apoptosis-inducing factor (AIF) caspase-independent apoptotic pathway in a normotensive Wistar Kyoto (WKY) group, a spontaneously early aged hypertensive (SHR) group and a hypertension with 12 weeks exercise training (SHR-EX) group.** (**A**) The 3 representative protein levels of AIF and EndoG extracted from the cerebral cortex of excised brain in WKY, SHR, and SHR-EX groups were measured by Wester blotting analysis. The α-tubulin was used as an internal control. (**B**) Bars represent the relative fold changes of protein quantification relative to the control group in EndoG and AIF proteins on α-tubulin and mean values ± SD (n=6 in each group). *: *p*<0.05, **: *p*<0.01 in comparison between SHR group and WKY rat group; #: *p*<0.05, ##: *p*<0.01 in comparison between SHR-EX group and SHR group.

To determine the effect of exercise training on the neural EndoG/AIF-related caspase-independent apoptotic pathway in the cerebral cortex, the protein level of Endo G and AIF in the three groups (WKY, SHR, and SHR-EX) were measured by western blot. The protein levels of EndoG and AIF in SHR were increased compared to WKY group. The protein levels of EndoG and AIF in SHR-EX group were decreased compared to SHR group, suggesting that exercise training could prevent neural apoptosis in the early aged hypertensive brain via EndoG and AIF pathways (Figure 2).

### Upstream components of neural Fas/FasL-mediated caspase-dependent apoptotic pathway

**Figure 3:**
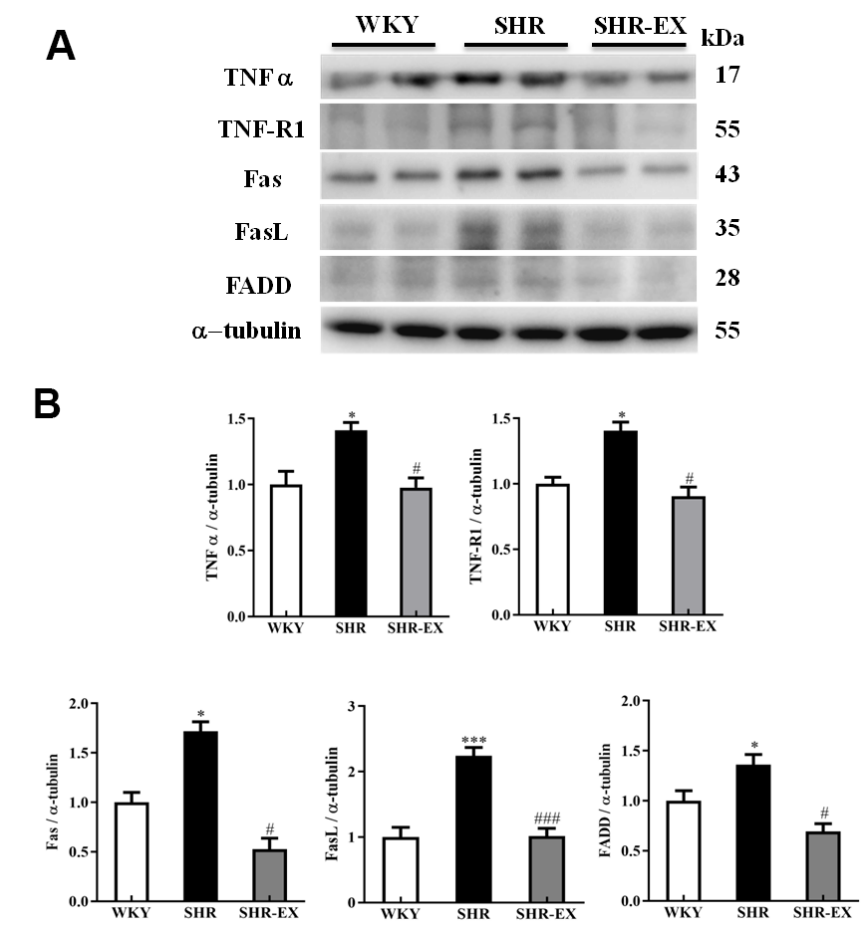
**The upstream components of Fas/FasL-mediated caspase-dependent apoptotic pathway in a normotensive Wistar Kyoto (WKY) group, a spontaneously early aged hypertensive (SHR) group and a hypertension with 12 weeks exercise training (SHR-EX) group.** (**A**) The representative protein levels of TNF α, TNF-R1, Fas receptor (Fas), Fas ligand (FasL), and Fas-associated death domain (FADD), extracted from the cerebral cortices of excised brain in WKY, SHR, and SHR-EX groups were measured by western blot analysis. The α-tubulin was used as an internal control. (**B**) Bars represent the relative fold changes of protein quantification relative to the control group in TNF α, TNF-R1, Fas, FasL, and FADD on α-tubulin and mean values ± SD (n=6 in each group). *: *p*<0.05, ***: *p*<0.001 in comparison between SHR group and WKY rat group. #: *p*<0.05; ###: *p*<0.001, in comparison between SHR-EX group and SHR group.

To investigate whether exercise training can affect the neural Fas/FasL-mediated caspase-dependent apoptotic pathway, western blot was conducted to measure the protein level of Fas, FasL, and FADD from the excised cerebral cortex tissues of the WKY, SHR and SHR-EX group. The levels of TNF α, TNF-R1, Fas, FasL, and FADD in SHR were increased compared to WKY group and those were decreased in SHR-EX compared with SHR group (Figure 3).

### Upstream components of neural mitochondria-mediated caspase-dependent apoptotic pathway

**Figure 4:**
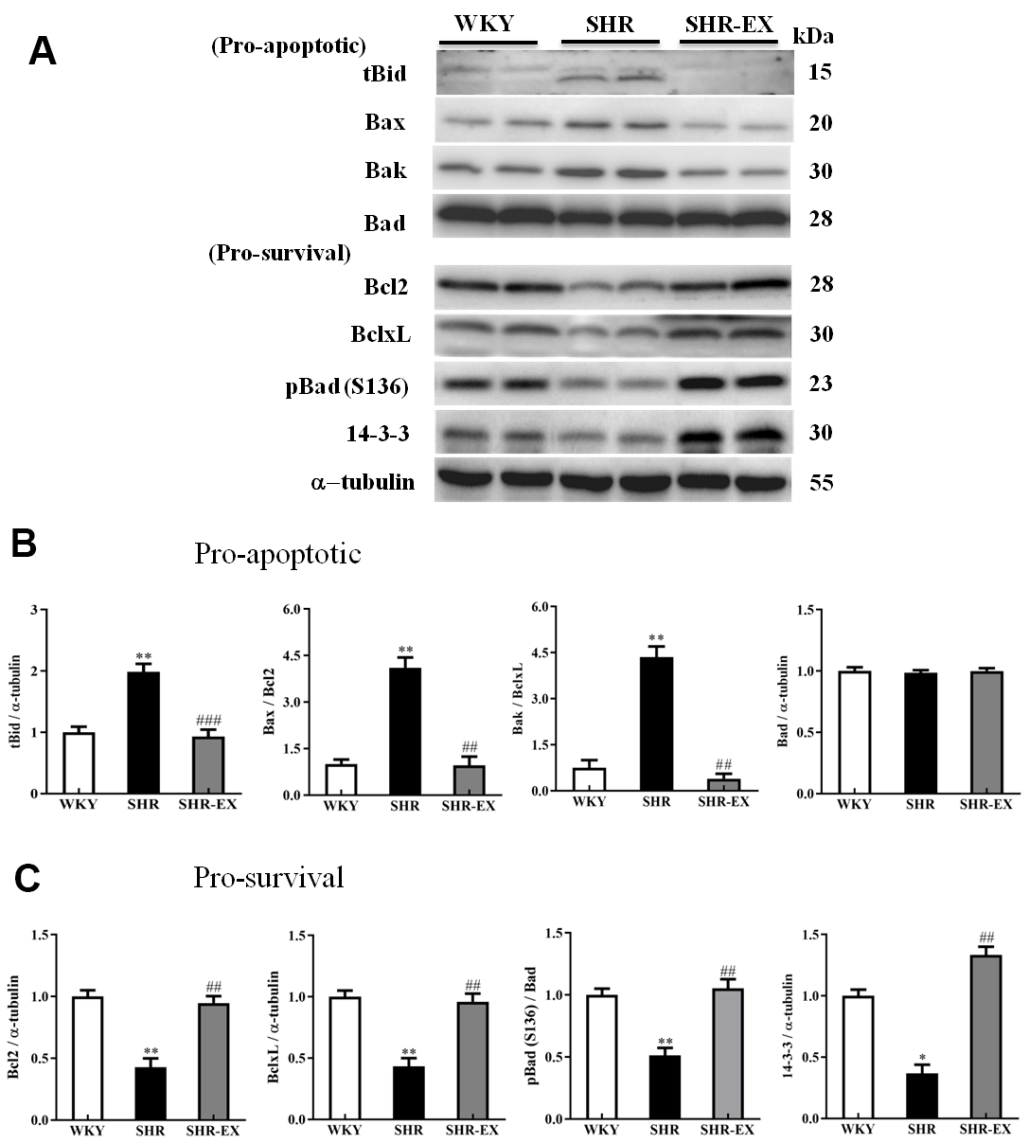
**The upstream components of mitochondria-mediated caspase-dependent apoptotic pathway in a normotensive Wistar Kyoto (WKY) group, a spontaneously early aged hypertensive (SHR) group and a hypertension with 12 weeks exercise training (SHR-EX) group.** (**A**) The representative protein levels of t-Bid (BH3 interacting domain death agonist), Bax (Bcl-2 Associated X), Bak (Bcl-2-agonist/killer 1), Bad (Bcl-2 agonist cell death) as well as Bcl-2, Bcl-xL, p-Bad, and 14-3-3 proteins extracted from western blotting analysis. The α-tubulin was used as an internal control. (**B**, **C**) Bars represent the cerebral cortices of excised brain in WKY, SHR, and SHR-EX groups were measured by Wt the pro-apoptotic as well as pro-survival relative fold changes of protein quantification relative to the control group in t-Bid, Bax/Bcl-2, Bak/Bcl-xL as well as pBad/Bad pBad and 14-3-3 on α-tubulin, respectively, and mean values ± SD (n=6 in each group). *: *p*<0.05, **: *p*<0.01, in comparison between SHR and WKY rat group; ##: *p*<0.01, ###: *p*<0.001 in comparison between SHR-EX group and SHR group.

To examine the upstream components of the neural mitochondria-mediated caspase-dependent apoptotic pathway consisting of both pro-apoptotic and anti-apoptotic of the Bcl-2 protein family, western blot analysis was conducted in the cortical tissues of the three groups including WKY, SHR, and SHR-EX. The levels of tBid, Bax, Bak, Bax/Bcl2 and Bax/Bcl-xL were higher in SHR group compared with WKY group as well as drastically lower in SHR-EX group than those in SHR group. The levels of Bcl2, Bcl-xL, pBad, pBad/Bad, 14-3-3 in SHR group were significantly decreased compared with WKY group as well as those were significantly increased in SHR-EX group compared with SHR group, indicating that exercise training inhibited neural mitochondria-mediated pro-apoptotic pathway and enhanced mitochondria-mediated anti-apoptotic pathway through these upstream components of mitochondria-mediated apoptotic pathway (Figure 4).

### The downstream components of neural Fas/FasL-mediated and mitochondria-mediated caspase-dependent apoptotic pathway

**Figure 5:**
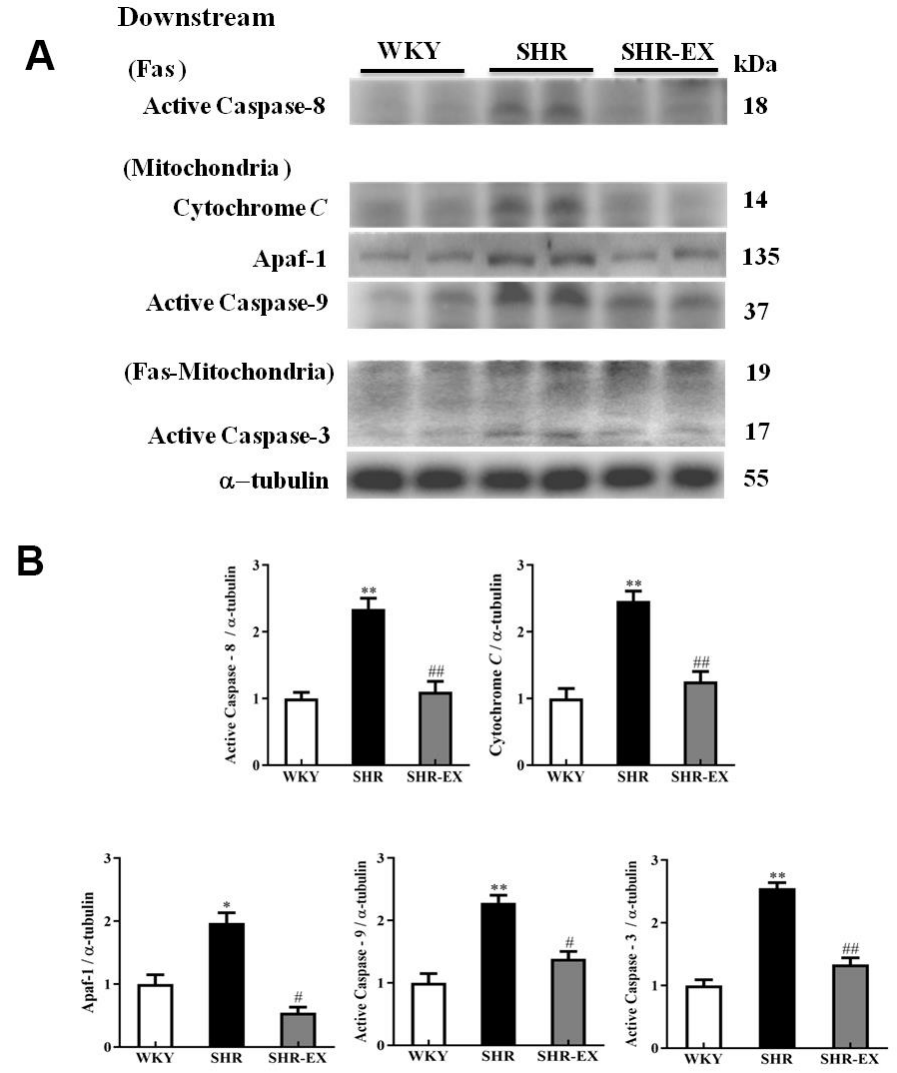
**The downstream components of Fas/FasL-mediated and mitochondria-mediated caspase-dependent apoptotic pathways in a normotensive Wistar Kyoto (WKY) group, a spontaneously early aged hypertensive (SHR) group and a hypertension with 12 weeks exercise training (SHR-EX) group.** (**A**) The representative protein levels of active Caspase-8 (Fas downstream), Cytochrome *c*, Apaf-1, active Caspase-9 (mitochondrial downstream), active Caspase-3 (Fas and Mitochondrial downstream) extracted from the cerebral cortex of excised brain in WKY, SHR, and SHR-EX groups were measured by Wester blotting analysis. The α-tubulin was used as an internal control. (**B**) Bars represent the relative fold changes of protein quantification relative to the control group in active Caspase-8, Cytochrome *C*, Apaf-1, active Caspase-9, active Caspase-3 on α-tubulin and mean values ± SD (n=6 in each group). *: *p*<0.05, **: *p*<0.01 in comparison between SHR group and WKY rat group; #: *p*<0.05, ## *p*<0.01 in comparison between SHR-EX group and SHR group.

The downstream components of Fas and mitochondria-mediated caspase-dependent apoptotic pathway in the cerebral cortex in WKY, SHR, and SHR-EX were investigated by western blot. The protein levels of active Caspase-8 and active Caspase-3 (apoptotic pathway), cytosolic Cytochrome, Apaf-1, active Caspase-9, active Caspase3 (mitochondria-mediated caspase-dependent apoptotic pathway) in SHR group were increased compared with WKY group whereas those components were decreased in SHR-Ex group compared with SHR group, suggesting that exercise training could prevent neural apoptosis through these downstream components of Fas/FasL-mediated and mitochondria-mediated caspase-dependent apoptotic pathway (Figure 5).

### The neural IGF-1-related pro-survival pathway

**Figure 6:**
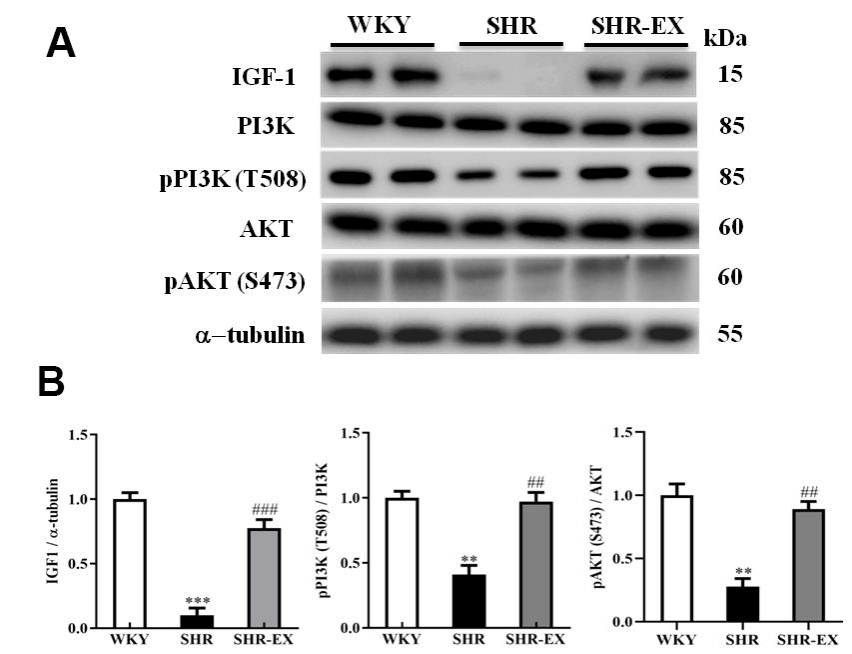
**The IGF-1-related survival pathway in a normotensive Wistar Kyoto (WKY) group, a spontaneously early aged hypertensive (SHR) group and a hypertension with 12 weeks exercise training (SHR-EX) group.** (**A**) The representative protein levels of IGF-1, PI3K, p-PI3K, AKT and p-AKT protein extracted from the cerebral cortex of excised brain in WKY, SHR, and SHR-EX groups were measured by western blot analysis. The α-tubulin was used as an internal control. (**B**) Bars represent the relative changes of protein quantification in IGF-1, p-PI3K/PI3K, and pAKT/AKT on α-tubulin and mean values ± SD (n=6 in each group). **: *p*<0.01, ***: *p*<0.001, in comparison between SHR group and WKY rat group; ##: *p*<0.01, ###: *p*<0.001, in comparison between SHR-EX group and SHR group.

To explore whether exercise training enhances the pro-survival components of neural apoptosis in the early aged hypertensive cortex, western blot was conducted to measure the protein level of IGF-1, PI3K, pPI3K, AKT and pAKT in the cerebral cortex. The expression levels of IGF-1, PI3K, pPI3K, AKT, and pAKT in SHR group were significantly decreased compared with WKY group. A significant increase of IGF-1, PI3K, pPI3K, AKT, and pAKT in SHR-EX group was observed compared with SHR group (Figure 6).

## DISCUSSION

### Major findings

**Figure 7:**
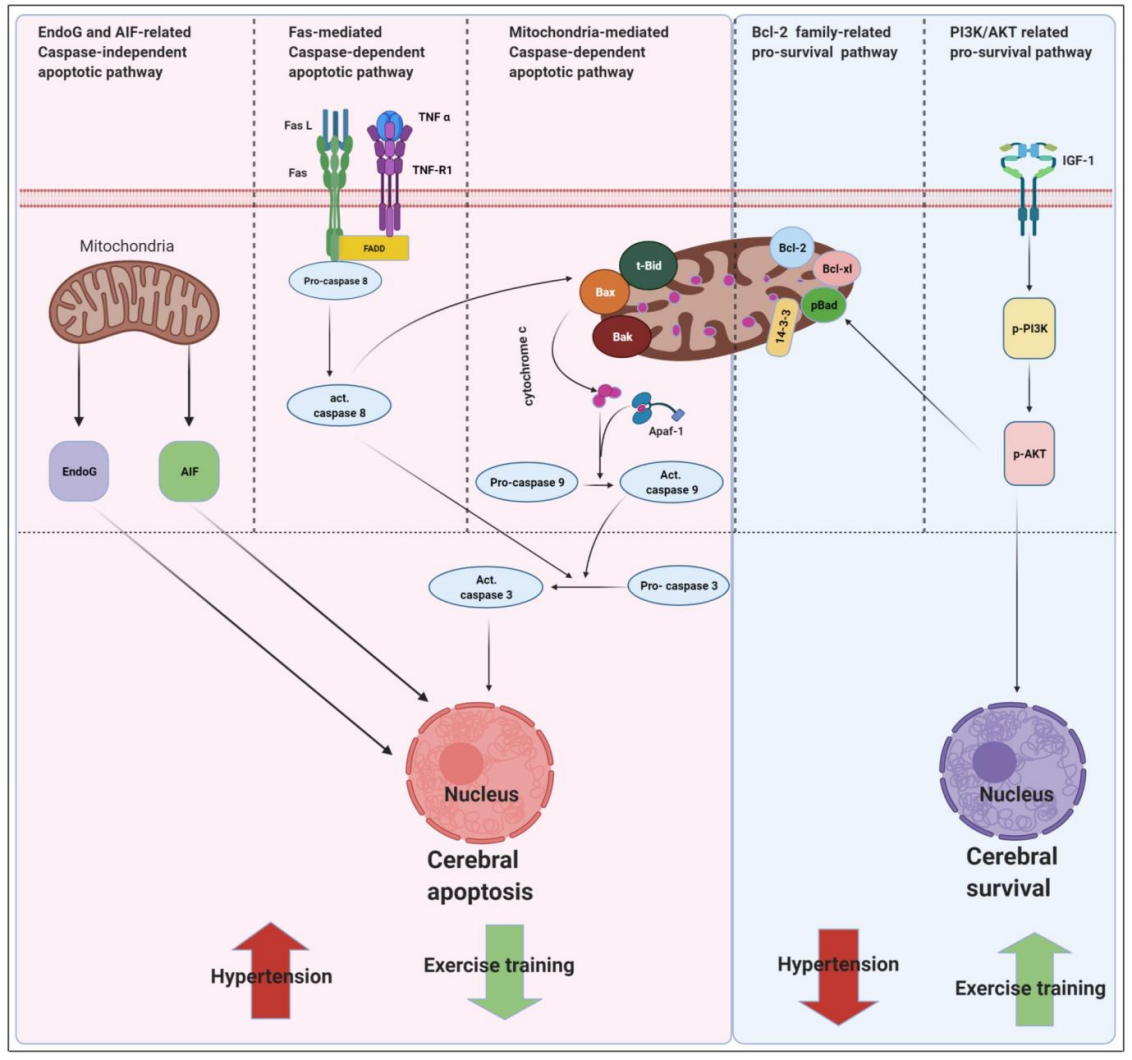
**Proposed hypothesis indicating that early aged hypertension appear to activate the EndoG/AIF-related caspase-independent, Fas/FasL-mediated caspase-dependent apoptotic pathways (Fas ligand, Fas receptor, TNF-α, TNF receptor 1, Fas-associated death domain, active Caspase-8 and active Caspase-3) and mitochondria-mediated caspase-dependent apoptotic pathway (t-Bid, Bax, Bak, Bad, cytochrome *c*, Apaf-1, active Caspase 9 and active Caspase-3) as well as suppresses Bcl-2 family-related pro-survival pathway (Bcl2, Bcl-xL, p-Bad, 14-3-3) and IGF-1 related pro-survival pathway (IGF-1, pPI3K/PI3K, and pAKT/AKT).** Whereas, exercise training tends to inhibits early aged hypertension-induce neural EndoG/AIF related caspase-independent, hypertension-induce Fas/FasL-mediated apoptotic and hypertension-induce mitochondria-mediated caspase-dependent apoptotic pathway as well as enhances Bcl-2 family-related pro-survival and IGF-1-related pro-survival pathway.

The main new findings were outlined as follows: 1) Early aged hypertension-activated neural EndoG/AIF-related caspase-independent, Fas/FasL-mediated caspase-dependent, and mitochondria-mediated caspase-dependent apoptotic pathways as well as suppressed Bcl-2 family-related and IGF-1-related pro-survival pathways in the cerebral cortex. 2) Exercise training decreased early aged hypertension-induced TUNEL positive apoptotic cells in the cerebral cortex. 3) Exercise training decreased early aged hypertension hypertension-induced neural EndoG/AIF-related caspase-independent apoptotic pathways, which was supported by the expression levels of EndoG and AIF in the cerebral cortex. 4) Exercise training reduced early aged hypertension hypertension-induced neural Fas/FasL-mediated caspase-dependent apoptotic pathway and the effect was confirmed by the reduction in expression levels of FasL, Fas, TNF-α, TNF receptor 1, FADD, active Caspase-8, and active Caspase-3 in the cerebral cortex. 5) Exercise training attenuated early aged hypertension hypertension-induced neural mitochondria-mediated caspase-dependent apoptotic pathway, as indicated by the decreases in expression levels of Bax, Bak/Bcl-xL, tBid, Apaf-1, Cytochrome c, active Caspase-9, and active Caspase-3 in the cerebral cortex; 7) Exercise training enhanced Bcl-2 family-related pro-survival protein levels (Bcl-2, Bcl-xL, pBad, 14-3-3) and IGF-1- related pro-survival protein levels (IGF-1, pPI3K/PI3K, pAKT/AKT) in the early aged hypertensive cerebral cortex. Taking our findings with the previously apoptotic theories together, we drew the hypothesized diagram (Figure 7) which suggested that cerebral cortex EndoG/AIF-related caspase-independent, Fas/FasL-mediated caspase-dependent and mitochondria-mediated caspase-dependent apoptotic pathways were augmented by early aged hypertension and were attenuated by exercise training. In contrast, the cerebral cortex Bcl-2 family-related and IGF-1-related pro-survival pathways were suppressed by early aged hypertension and were enhanced after exercise training. Exercise training not only has the neuroprotective effects through anti-apoptotic and pro-survival pathways, but also may have other indirect beneficial effects from multiple systems such as enhancing insulin sensitivity, anti-oxidative stress, anti-inflammation, hormonal balance, neuromodulatory balance, decreasing neurotoxicity, improving neuronal mitochondrial function or other unclear interaction factors [16].

Hypertension is a devastating vascular risk factor for end-organ damage [34]. Increased apoptosis in hypertensive organs such as the heart (ventricular cardiomyocytes), kidney (inner cortex and medulla), and brain (cortex, striatum, hippocampus, and thalamus) was observed with extensive effect of cell death inducer [35]. Unregulated neural cell death in the cerebral cortex could be detrimental as it could deteriorate neural function and this sequester of uncontrolled cell death could be influenced in certain diseases. Hypertension induced a neural loss in the brain which would further aggravate blood flow reduction and impaired function resulting in end-stage brain damage [36]. Evidence revealed that hypertension extended ischemic brain lesions and was progressively observed to coexist with neurodegenerative illness like Alzheimer's Disease [37]. Since hypertension is the main reason for the increase of neural loss and deterioration of brain performance, therefore evaluating the degree of neural apoptosis at early stage of hypertension might be significant to minimize the subsequent complications.

Exercise training is a significant lifestyle modification for improving general body health. The benefit of exercise training in hypertension is determined by changes in the peripheral and central mechanisms of blood pressure control [11]. A previous study showed that low intensity of exercise training substantially decreased systolic blood pressure, with high chances of reducing the diastolic blood pressure in stage one-hypertension [38]. In this present study, the systolic blood pressure, diastolic blood pressure and mean blood pressure in early aged hypertensive rats were decreased after 12 weeks of exercise training on a treadmill. Base on the results of this study, we reconfirmed that exercise training could be considered as an alternative or adjunct to treat hypertension as well as an adjuvant to prevent hypertension-induced complications.

Both human and animal studies had substantiated that exercise training could effectively improve brain function and provide neuroprotection [39, 40]. Substantial reduction of AIF expression or suppressing AIF activity provided neuroprotection against intense neurodegeneration [41]. In this present study, exercise training was found to reduce hypertension-induced EndoG/AIF-related caspase-independent apoptotic pathways in the cerebral cortex which was evidenced by reduced expression levels of EndoG and AIF. No previous study had showed exercise training prevents neural EndoG and AIF in hypertensive brain, but one animal study reported that exercise training prevented neural cell death with a significant reduction of apoptosis-inducing factor (AIF) in the brain undergoing ischemia and reperfusion [25]. The suppression of EndoG and AIF protein in the early aged hypertensive cerebral cortex by exercise training was first reported in this study.

Our present study demonstrated that exercise training played a significant role in inhibiting hypertension-induced Fas/FasL-mediated caspase-dependent apoptotic pathway in the cerebral cortex, as indicated by decreased expression levels of FasL, Fas, FADD, active Caspase-8, and active Caspase-3. A previous study reported that swimming exercise on D-galactose-induced aging rats decreased protein Fas ligand, Fas, FADD and active Caspase-8 expression in the hippocampus, which ultimately suppressed apoptosis [33]. Another evidence postulated that significant down-regulation of Fas receptor was neuroprotective and could reduce posttraumatic axonal degeneration after acute spinal injury [42]. Primarily, exercise training decreased activities of Fas/FasL-mediated caspase-dependent apoptotic pathway in the cerebral cortex of early aged hypertensive rat was preliminarily revealed in this study.

Neural mitochondria-mediated caspase-dependent apoptotic pathway responding to apoptosis depends on the balance between the pro-apoptotic and anti-apoptotic factors [43]. A previous study revealed that pre-exercise suppressed pro-apoptotic B-cell lymphoma 2 (Bcl-2) family molecules such as Bid in traumatic brain injury, diminished mitochondrial permeabilization with prevented release of Cytochrome c, and decreased migration of apoptosis-inducing factor (AIF) to the nucleus and prevented caspase activation [19]. In this current study, exercise training was found to attenuate hypertension-induced neural mitochondria-mediated caspase-dependent apoptotic pathway. This corroborated finding was based on decreased expression levels of Bax/Bcl-2, Bak/Bcl-xL, tBid, Apaf-1, Cytochrome *c*, active Caspase-9, and active Caspase-3 in the cerebral cortex after 12 weeks of exercise training. A previous study showed that physical exercise improved brain cortex and cerebellum through reduction of pro-apoptotic Bax/Bcl-2 proportion [16].

The recovery of neurological disorders mainly relies on neural cellular survival [25]. Former research indicated that exercise training simultaneously suppressed cell death pathways and enhanced survival pathways in brain subjected with ischemia/reperfusion injury as well as increased expression of Bcl-xL proteins [25]. Increased anti-apoptotic activities reduced neuronal loss in brain ischemia [44]. Interestingly, in this investigation, exercise training was observed to enhance Bcl-2 family-related pro-survival protein levels (Bcl-2, Bcl-xL, pBad, 14-3-3) and IGF-1-related pro-survival protein levels (IGF-1, p-PI3K/PI3K, pAKT, AKT) in the early aged hypertensive cerebral cortex, suggesting another possible therapeutic approach. The increased expression of pPI3K, pAKT and Bcl-xL in the hippocampus of aging rats undergoing swimming was found essential to prevent neural apoptosis [33]. Four weeks of moderate-intensity exercise training on aging mice was found to induce 14-3-3 protein, heat shock proteins 70 and increase related neurogenesis biomarkers in the hippocampus with significant effect of enhancing neuroprotection, neurogenesis and synaptic strength [45]. Moreover, exercise training increased optimal blood flow and activated growth factors in traumatic brain injury and improved brain function via promoting neurogenesis, angiogenesis and synaptogenesis [46]. The effects of exercise training on Bcl-2 family-related pro-survival protein and IGF-1-related pro-survival protein in the early aged hypertensive cerebral cortex were first investigated in this study.

## Conclusion

This present study showed that twelve weeks of exercise training prevented neural apoptosis in early aged hypertensive rat cerebral cortex by suppressing the activation of pro-apoptotic pathways and enhancing pro-survival pathways. Based on the former studies, there was no similar study to support the recent finding in early aged hypertensive rat cerebral cortex. The results of this study suggested that exercise training might have significant benefits on the brain. In general, the study hereby supported the therapeutic abstract to advance the significance of exercise training for treating and preventing neurological abnormalities associated with hypertension.

### Perspectives

Hypertension is regarded as a critical vascular risk factor for developing cognitive impairment and brain damage. A disease modifying approach for the treatment and prevention of abnormal brain function in hypertension is desperately needed. Apart from antihypertensive medications, exercise training would be considered as the most rational approach for enhancing neuroprotection and neurorestoration of hypertension. Base on the result of this study, hypertension was known to induce neural apoptosis in the cerebral cortex and was attenuated by exercise training. Therefore, it is essential to realize the possibility for generating brain abnormalities in aged hypertension as well as the significant of exercise training as a therapeutic approach for preventing brain degeneration in aged hypertension. Since it is strenuous to extract cerebral tissues from human brain, this current animal demonstration might help to provide a panorama and elucidation of the mechanism on how exercise training attenuates brain damage or neural apoptosis related to neurological disorders in human. The study explicates the effect of 12 weeks of exercise training against early aged hypertension-induce neural apoptosis in the cerebral cortex through suppressing pro-apoptotic pathways and activating anti-apoptotic activities as well as enhancing the pro-survival pathway, which may aid in preventing other neurological conditions resulting in brain damage. Further clinical studies are needed to elucidate any feasible therapeutic approach in human.

## MATERIALS AND METHODS

### Animal

The experiment was performed on normotensive Wistar Kyoto rats (WKY, n=10) and spontaneously early aged hypertensive male rats (SHR, n=20) at 12 months old, males. The spontaneously early aged hypertensive rats (SHR) were further divided equally into sedentary (SHR) group and exercise (SHR-EX) group. The rats were housed under a 12-h light-dark cycle to maintain good living conditions. Standard laboratory chow (Lab Diet 5001; PMI Nutrition International, Brentwood, MO, USA) and water ad libitum were used for feeding the rats. The protocol applied for the animal caring was following The National Institutes of Health Guide for the Care and Use of Laboratory Animals and approved by the committee of China Medical University Animal Center, Taichung.

### Exercise training

A motor-driven levelled treadmill (Model T408E, Diagnostic and Research Instruments Co., Taoyuan, Taiwan) was used for animal exercise. On the first day, only SHR-EX group was prepared to run at 15 m/min for 20 min in order to develop habitual acquaintance with the training. The running period was gradually increased by 10 min each day until it reached a running period of 60min/day within five days for which habitual acquaintance of training was achieved. During the training period, we placed all the three groups on the treadmill. The SHR-EX group run at a speed of 18m/min then gradually accelerated at a speed of 3 m/min in every 2 weeks up to 27 m/min. The SHR-EX group was trained at 60min in each day for 5 days a week totaling for 12 weeks. Whereas, the other groups (WKY and SHR) were assigned to run without undergoing similar environmental stimulation as SHR-EX group.

### Measurement of blood pressure

At rest, systolic, diastolic and mean arterial blood pressure of the animals were measured applying the Indirect Tail cuff method (BP98A, Softron, Tokyo, Japan) once a week before conducting the sacrifice.

### Citrate synthase activity

The effectiveness of exercise training was confirmed by increased in Citrate Synthase Activity. We measured the Citrate Synthase Activity by using homogenized soleus muscle samples (in five volumes of 0.1Mof Tris buffer containing 0.1%Triton X-100) from all the three groups (WKY, SHR and SHR-EX). We used spectrophotometric readings at 412 nm (UV-240, Shimadzu Co., Tokyo, Japan). All samples were tested in duplicate.

### Preparation of brain tissue

The rats were first put under anaesthesia to minimize suffering. Isoflurane solution (2% concentration) delivered through carbogen gas (95% O_2_ and 5% CO_2_) was used for anesthetizing the rats by inhalation. After being sacrificed, the brain tissues were removed and cleaned with cold ice of 0.9 % Sodium chloride (NaCl) and weighed, then placed in a 4% (w/v) solution of PFA for 15 min, transferred to a 20% (w/v) fresh sucrose solution for 12-16 hours, and then retransferred to a 30% (w/v) fresh sucrose solution for 12-16 hours. The brain tissues were sliced into the cerebral cortex section and tested by DAPI /TUNEL staining and western blot.

### TUNEL assay and DAPI staining

After the decapitation, the cut brain pieces were soaked in neutral buffered formalin (NBF) then deparaffinized with Xylene. Graded alcohols were used to rehydrate the deparaffinized sections. After being soaked in 200 ml 0.1M Citrate buffer (pH 6, with the 750W (high) microwave irradiation) for 1 min, the sections were blocked in the buffer for 30 min and washed twice in PBS. The tissue sections were mounted for TUNEL assay (Terminal deoxynucleotidyl transferase and fluorescein isothiocyanate-dUTP assay) using an apoptosis detection kits (Roche Applied Science, Indianapolis, IN, USA) for 60 min, 37° C as well as adding DAPI Fluoromount G for 15 min at 18-25° C. For fluorescence evaluation by using an Inverted Microscope (Eclipse Ti-U, NIKON, Japan), the excitation wavelength in the range of 450-500 nm and detection in the range of 515-565 nm (green) and 358-461 (blue) were used. We determined the number of TUNEL-positive cells and the DAPI-stained nuclei on different separated fields (like 3 fields/sample and 3 samples/group) of the cerebral cortex in random selections and repeated the process for at least nine times. To avoid bias and ensure all results are reliable, two independent individuals conducted the counting separately.

### Electrophoresis and western blot

Bradford method (Bio-Rad Protein Assay, Hercules, CA, USA) was applied to measure the protein concentration of neural tissues obtained from the cerebral cortex. Protein samples (100μg/lane) were divided on a 12% SDS-PAGE (sodium dodecyl sulfate-polyacrylamide gel electrophoresis) at a voltage of 80V for 20 minutes then at 100V for 2 hours. After the electrophoresis, proteins tissues were transferred to polyvinylidene difluoride (PVDF) membranes (Millipore, Bedford, MA, USA) at a constant voltage of 100V for 1.5hours. 5% of non-fat milk was used for one hour to block PVDF membranes. The membranes were incubated overnight at 4° C with a diluted solution mixed with primary antibodies including IGF-1, AKT, pAKT (S473), PI3K, Bcl-2, Bcl-xL, pBad (S136), Bad, Bax, Bak, Cytochrome *c*, TNF α, TNF-R1, FasL, Fas receptor, FADD, Apaf-1, active Caspase-8, active Caspase-9, active Caspase-3 with 1:1000 dilution (Cell Signalling Technology, Beverly, MA, USA) and 14-3-3, pPI3K (T508), EndoG, AIF, α-tubulin with 1:500 dilution (Santa Cruz Biotechnology, Santa Cruz, CA) were incubated in antibody binding buffer at 4° C overnight, washed by TBST, incubated with an HRP-conjugated second antibody solution diluted at 1:5000 (Santa Cruz Biotechnology, Santa Cruz, CA, USA) at room temperature, and then washed in the TBST buffer. With an enhanced chemiluminescence (ECL) Western blot reagent (Millipore Corporation, Bedford, MA), the immunoblotted proteins were visualized and quantified by a chemiluminescence detection system (Fujifilm LAS-3000, Fuji, Tokyo, Japan). The density of the bands was quantified by densitometry using Gel-Pro Analyzer densitometry software (Media Cybernetics, Silver Spring, MD, USA).

### Statistical analysis

All data including brain weights, level of proteins, and percentage of TUNEL-positive cells were compared among control (WKY), sedentary (SHR) and exercise (SHR-EX) group, respectively. One-way analysis of variance (one-way ANOVA) statistical technique was applied to perform pre-planned contrast comparison for negative or positive control. SPSS 22.0 software was used for analyzing and *p*<0.05 was regarded as significant.
